# Cortical interneurons from human pluripotent stem cells: prospects for neurological and psychiatric disease

**DOI:** 10.3389/fncel.2013.00010

**Published:** 2013-03-13

**Authors:** Charles Arber, Meng Li

**Affiliations:** ^1^Stem Cell Neurogenesis, MRC Clinical Sciences Centre, Imperial College LondonLondon, UK; ^2^School of Bioscience, School of Medicine, Neuroscience and Mental Health Research Institute, Cardiff UniversityCardiff, UK

**Keywords:** cortical interneurons, embryonic stem cells, cell therapy, proof-of-principle fetal transplant, seizure

## Abstract

Cortical interneurons represent 20% of the cells in the cortex. These cells are local inhibitory neurons whose function is to modulate the firing activities of the excitatory projection neurons. Cortical interneuron dysfunction is believed to lead to runaway excitation underlying (or implicated in) seizure-based diseases, such as epilepsy, autism, and schizophrenia. The complex development of this cell type and the intricacies involved in defining the relative subtypes are being increasingly well defined. This has led to exciting experimental cell therapy in model organisms, whereby fetal-derived interneuron precursors can reverse seizure severity and reduce mortality in adult epileptic rodents. These proof-of-principle studies raise hope for potential interneuron-based transplantation therapies for treating epilepsy. On the other hand, cortical neurons generated from patient iPSCs serve as a valuable tool to explore genetic influences of interneuron development and function. This is a fundamental step in enhancing our understanding of the molecular basis of neuropsychiatric illnesses and the development of targeted treatments. Protocols are currently being developed for inducing cortical interneuron subtypes from mouse and human pluripotent stem cells. This review sets out to summarize the progress made in cortical interneuron development, fetal tissue transplantation and the recent advance in stem cell differentiation toward interneurons.

## Introduction

The complex circuitries of the cerebral cortex comprise networks produced by two major neuronal cell types: the excitatory glutamatergic projection neurons (Pyramidal cells) and gamma-aminobutyric containing (GABAergic) interneurons. Pyramidal neurons are the primary neural cells that specialize in transmitting information between different cortical regions and to other regions of the brain. Interneurons on the other hand represent a minority (~20%) of the entire neocortical neuronal population (Whittington and Traub, 2003; Wang et al., 2004; Hensch, 2005). GABAergic interneurons are highly heterogeneous and had been overlooked for many years due to their vast complexities. However, it is thought that they play a vital role in the function of the cerebral cortex. Interneurons provide inhibitory inputs that shape the responses of pyramidal cells and prevent runaway excitation. They regulate the timing and synchronization of population rhythms expressed as cortical oscillations (Haider et al., 2006; Klausberger and Somogyi, 2008). Consequently, reduction in tonic inhibition through interneuron hypoplasia in the cortex has been implicated in major neurological and psychiatric illness, including epilepsy, mental retardation, autism, and schizophrenia (Powell et al., 2003b; Lewis et al., 2005; Akbarian and Huang, 2006; Yizhar et al., 2011).

Interneurons exhibit a remarkable intrinsic ability to migrate and therefore offer a potential neuronal source for cell-based therapies for treating the above mentioned interneuron deficiencies. Recently, several elegant works have shown that transplanting fetal interneuron precursors can create a new critical period of plasticity in the recipient brain and reduce seizures in animal models of epilepsy (Wichterle et al., 1999; Alvarez-Dolado et al., 2006; Baraban et al., 2009). Interneuron transplants can also reduce movement deficits in a rat model of Parkinson's disease (Martinez-Cerdeno et al., 2010). When transplanted into the spinal cord, interneurons also help decrease pain sensation (Braz et al., 2012). Together, these proof of principal studies raise hope for the possibility of using neuronal transplantation to treat diseases like epilepsy and Parkinson's.

In this review, we provide a brief overview of the development and function of cortical interneurons. We then evaluate the current progress of interneuron production from pluripotent stem cells. Finally, we discuss the potential and the challenges of exploiting stem cell technologies for modeling neurological and neuropsychiatric disorders, drug discovery and cell therapy.

## Classifications of cortical interneurons

Cortical interneurons are cells that connect only with nearby neurons, to distinguish them from “projection” neurons, whose axons span to more distant regions of the brain. Interneurons typically express the inhibitory neurotransmitter gamma-amino butyric (GABA) and have aspiny dendrites (Markram et al., 2004). Despite these common features, interneurons display huge diversity in morphology, physiology, and marker expression. Here we provide a brief summary of the complex classification of cortical interneurons. For a thorough evaluation of the subject, refer to the excellent reviews by Ascoli et al. (2008) and Batista-Brito and Fishell (2009).

Efforts to distinguish interneurons based on morphology have led to complex conclusions. Classifications based on synaptic targets of interneurons, i.e., axon, soma, proximal dendrite, or distal dendrite, provide a useful starting point (DeFelipe, 1997). This classification defines the role of the interneuron in the microcircuit. Interneurons that form synapses on pyramidal cell axons have the potential to eradicate action potential transmission; conveying power to this subtype after sensing the global excitatory environment. This is in contrast to interneurons that contact distal dendrites of pyramidal cells that may influence signal formation in more subtle ways, e.g., necessitating summation of input signals (Markram et al., 2004). Interneuron groups that have dendritic connections are more numerous and diverse than those on the soma and axons due to the intricacies involved in these roles.

It should be noted that not all GABAergic neurons in the cortex are local interneurons with short-range associations. Approximately 0.5% of cortical GABAergic cells are “projection” neurons with long-range axons. The function of these cells in the mature cortex remains uncertain (Tamamaki and Tomioka, 2010).

The branching morphology of an interneuron and its cortical layer position can enable classifications into interneuron type. Some of these cells were first described by the monumental work by Cajal y Ramon over a century ago (DeFelipe, 2002; Sotelo, 2003). The groups include large basket cells, small basket cells, nest basket cells, chandelier cells, Martinotti cells, bitufted cells, bipolar cells, and double bouquet cells. This information is also relevant for the synaptic targets of the cells discussed above (Ascoli et al., 2008).

Interneurons can be classified based on their electrophysiological properties, explored in slice culture studies. Classes such as fast-spiking, non-adapting non-fast-spiking, adapting, irregular spiking, intrinsic burst firing, and accelerating have been described (Ascoli et al., 2008). It is important to note that the morphology and electrophysiological properties of the cells do not correlate directly, adding further complexity to the classification problem. In an elegant study, Toledo-Rodriguez and colleagues performed electrophysiological investigations followed by single cell expression analysis (Toledo-Rodriguez et al., 2004). Interestingly, they found individual genes that code ion channels which are predictive of electrophysiological class, for example *Kv1.1, HCN1*, and *Caα1A*; potassium, sodium and calcium channel proteins respectively. This may become critical for grouping interneurons into discrete subtypes.

Finally, interneurons can be distinguished based on expression of calcium binding proteins and neuropeptides. It has become convenient to distil the cells into three groups based on expression of Parvalbumin, Somatostatin, and Calretinin, which largely do not overlap (Kubota et al., 1994; Kawaguchi and Kubota, 1997). There are a number of other markers that may be used to distinguish interneuron subclasses. These include Calbindin, a calcium binding protein often used to describe cortical interneurons. Calbindin is coexpressed with Parvalbumin or Somatostatin in up to 80% of interneurons, making Calbindin an inappropriate marker (Kawaguchi and Kubota, 1997). Additionally, Neuropeptide Y (NPY) and Vasoactive Intestinal Polypeptide (VIP) are useful markers proteins, although these can display some colabelling with Calretinin positive cells. All of these expression markers may have little functional relevance; however, it has become clear from developmental biology that these classes originate in distinct locations, producing a useful fate map for developmental biologists. Again, there is no complete overlap between morphology, physiology and these expression-based classes.

The extensive variability of interneurons and the complexity in defining subclasses has led to the theory of an interneuron continuum, negating the presence of distinct subclasses (Parra et al., 1998). The study by Toledo-Rodriguez, demonstrating clustering of subgroups dependent upon voltage gate channels, argues against this continuum and encourages new techniques to classify subgroups. The field of stem cell biology utilizes information from developmental biology to mirror development *in vitro*. For this reason, it is convenient to employ developmental origin-based division in this review, i.e., Parvalbumin, Somatostatin, and Calretinin.

## Development of cortical interneurons

Although distributed dorsally in the cerebral cortex of the mature brain, cortical interneurons are derived from neural precursors generated in the ventral forebrain (telencephalon) and undergo major tangential migration to their dorsal target tissues. Fate mapping studies via isochronic, homotopic transplantation of labeled ventral forebrain tissues *in utero* and *in vitro* have demonstrated the vast migratory capacity of ventral progenitors and their ability to form GABAergic interneuron subtypes (Anderson et al., 1997; Lavdas et al., 1999; Nery et al., 2002).

**Figure 1:**
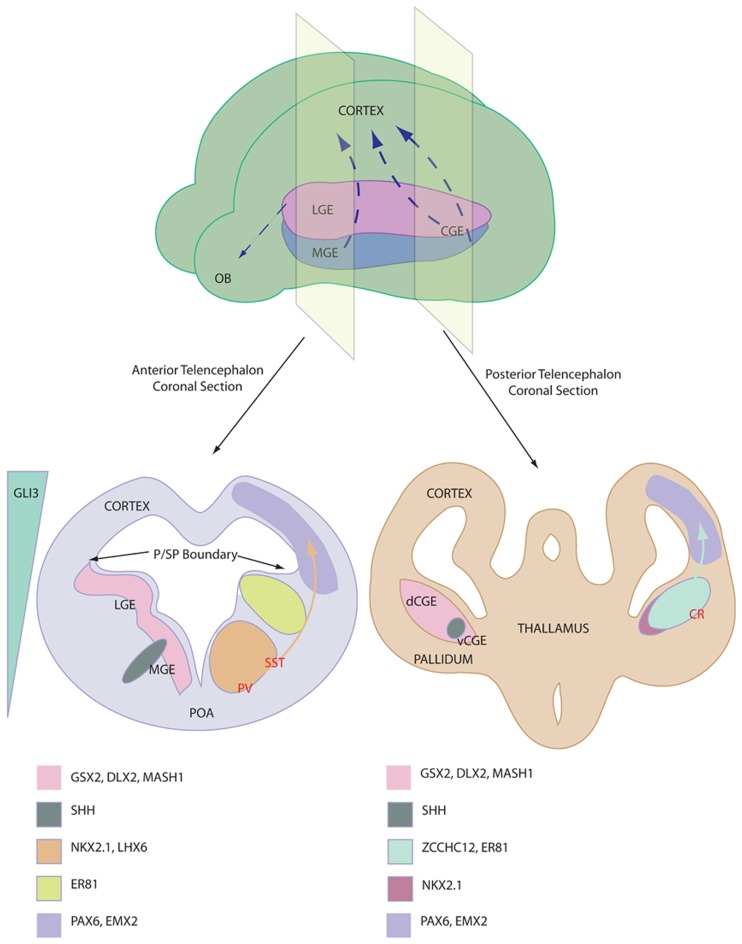
**Depiction of interneuron birth place and relevant gene expression profile. Top** shows ganglionic eminence distribution in a mouse brain and the major interneurons migration streams, NB CGE is a caudal extension of the more anterior tissues. **Bottom** shows anterior (left) and posterior (right) coronal sections through the telencephalon of an E13 mouse. Pertinent marker gene expression profiles are depicted of the proliferative niches of the ganglionic eminences. Relative origin of interneuron subtypes are shown in RED. Note the Shh expression domains and the reciprocal Gli3 expression. Abbreviations; LGE, lateral ganglionic eminence; MGE, medial ganglionic eminence; CGE, caudal ganglionic eminence (v ventral and d dorsal); OB, olfactory bulb; P/SP, pallial subpallial; POA, preoptic area; PV, Parvalbumin; SST, Somatostatin; CR, Calretinin.

The ventral telencephalon (also referred to as the subpallium) is divided into three neurogenic domains, the lateral- medial- and caudal-ganglionic eminences (LGE, MGE, and CGE respectively), see Figure 1. The LGE is the birthplace of the striatal projection neurons and a small population of olfactory bulb interneurons that migrate rostrally (Waclaw et al., 2009). The MGE and the CGE are the major sites of interneurogenesis, shown via the transplantation of labeled tissue (Xu et al., 2004; Butt et al., 2005). The CGE has been described as a caudal extension of the LGE and the MGE and as such the three tissues share many common gene expression profiles (Flames et al., 2007). For example *Gsx2, Dlx2*, and *Mash1* are three transcription factors involved in neurogenesis, patterning and migration and are expressed throughout the ganglionic eminences. Despite this similarity, there are genetic differences and precise expression domains that are starting to be described (Flames et al., 2007; Willi-Monnerat et al., 2008). Most importantly, the expression of *Nkx2.1*, a Shh responsive gene (Xu et al., 2010), defines the MGE and the ventral CGE, discerning these tissues from the LGE and the dorsal CGE (Sussel et al., 1999).

*Nkx2.1* expression is vital for MGE-based interneurogenesis, whereby its knockout reduced GABAergic populations in the cortex by 50% (Sussel et al., 1999). *Nkx2.1* has a critical function to induce *Lhx6*, a gene which is required for specification and migration of MGE-derived GABAergic interneurons (Du et al., 2008). After migrating to the cortex, these *Shh/Nkx2.1/Lhx6*-patterned progenitors mature *in situ* into Parvalbumin and Somatostatin expressing cortical interneurons. Interneuron specification is origin specific (Butt et al., 2005) and different regions of the MGE are thought to give rise to the two cell types. Somatostatin-expressing interneurons originate in the more lateral MGE, where Shh expression is higher, whereas Parvalbumin-expressing interneurons are derived from the more ventral MGE domain (Wonders et al., 2008; Xu et al., 2010; Inan et al., 2012). FGF signaling has also been implicated in ventral forebrain development, as receptor knockout leads to aberrant development (Storm et al., 2006). It should be noted that the MGE gives rise to other cell types, including striatal interneurons, cholinergic cells and glia, displaying distinct marker profiles.

Calretinin-expressing interneurons are unaffected by *Nkx2.1* knockout (Sussel et al., 1999) and were subsequently demonstrated to be derived in the dorsal CGE (dCGE) (Xu et al., 2004). These interneurons are dependent upon early expression of *Gsx2*, which is expressed higher in the dCGE and LGE compared with the MGE. Recent work has proposed a novel role for Activin signaling in inducing *Gsx2* and Calretinin-expressing interneurons in mouse and human embryonic stem cells (ESCs) (Cambray et al., 2012). Additionally, NPY-expressing cells are specified in the CGE in an undefined manner. Reports of the presence of Parvalbumin and Somatostatin immunoreactivity in the CGE can be attributed to progenitors migrating through the CGE from the MGE. In addition to the cortex, CGE-derived progenitors contribute strongly to interneuron populations in the hippocampus (Nery et al., 2002).

Once specified in the early subpallium, the interneuron progenitors migrate to their target tissues in the upper layers of the cortex or the hippocampus (Miyoshi and Fishell, 2011). This migration is dependent upon *Dlx1/2* and *Mash1* expression, as progenitors accumulate in the ventral forebrain in mouse knockout models of these genes (Long et al., 2009) and cortical tissues exhibit a 75% reduction in GABAergic cell numbers (Anderson et al., 1997). The intricacies of the interneuron migration are not well understood with a multitude of undefined factors directing this complex process (Faux et al., 2012). However, it appears that the cells do not follow corticofugal fibers toward target locations (Nery et al., 2002). Many contact the cortical ventricular zone together with newly-born cortical cells before migrating radially to their target laminae (Nadarajah et al., 2002). Interestingly, the different subtypes of cortical interneuron have differential target tissues, with respect to gross domains in the cortex as well as layer preference (Nery et al., 2002). Temporal differences in migration capacity have been described and cortical interneurons are believed to migrate in an “inside-out” pattern, with deep layer cells migrating earlier in development. This may have important implications on transplantation and is discussed below.

These above observations of cortical interneuron development have been based in rodent models. Recent data has reinforced the conserved nature of interneuron birth and migration in human development. For example, Calretinin progenitors have been shown to originate in the human basal forebrain (Jakovcevski et al., 2011; Zecevic et al., 2011) and loss of the ventral forebrain in the human fetus due to stroke leads to reduced numbers of Somatostatin and NPY-expressing interneurons (Fertuzinhos et al., 2009). These studies also indicated that there are critical differences between human and rodent development. Rodent interneurons do not express *Nkx2.1* during migration, whereas human progenitor cells were shown to maintain NKX2.1 expression into the cortex (Rakic and Zecevic, 2003). Here there is an additional mitotic event before the cells migrate radially to their final positions (Letinic et al., 2002). This may add to the complexity, size and additional cortical layering evident in the higher mammalian cortex and is a feature repeated in simian development (Petanjek et al., 2009). Therefore, generalization and inferences for human development based on murine data must be taken with caution; however, evidence does suggest the presence of many parallels between the two developmental systems.

Thus, interneurons are an extremely diverse population. Some diversity can be attributed to differential developmental origins, different morphogen responsiveness and fate determinant expression. This data becomes useful for stem cell biologists to parallel development *in vitro*.

## Interneuron dysfunction and diseases

Deficiencies in cortical interneuron numbers may arise from brain trauma, viral infections or gene mutations effecting function or migration; reviewed in Rossignol (2011). The “GABA hypothesis” states that a reduced level of tonic inhibition in the cortex or hippocampus leads to over-excitability and a spread of seizure (Cossart et al., 2001). Therefore, interneuron dysfunction has been associated with epilepsy, autism (a disease characterized by seizure) (Hussman, 2001), Tourette's syndrome, schizophrenia, and anxiety disorders (Lewis, 2000).

After initial epileptogenesis, there are a number of physiological alterations in the local cellular environment that contribute to chronic disease progression. This includes aberrant neurogenesis, alterations in cellular distributions, and gliogenesis (Maisano et al., 2009). One example is the recruitment of astrocytes to the focal site of seizure that express adenosine kinase; a molecule that degrades the anticonvulsant adenosine and potentiates chronic seizures (Gouder et al., 2004).

There are a number of animal models for seizure-based disease. Overstimulation via the chemoconvulsant substances kainic acid or pilocarpine leads to acute seizure and development of spontaneous seizures over time (Maisano et al., 2009). Additionally, there are a number of genetically altered paradigms that lead to interneuron deficiencies, many of which are neonatal lethal. Loss-of-function mutations of *Shh* or *Nkx2.1* lead to improper patterning of the ventral telencephalon and a lack of interneuron generation (Chiang et al., 1996; Sussel et al., 1999). *Dlx1/2* knockout mice exhibit a defect in migration (Long et al., 2009). Mutations of the homeobox gene *ARX* in human subjects have demonstrated an interneuron migration defect and patients display autism and epilepsy. *ARX* is regulated by *DLX2* (Friocourt and Parnavelas, 2010). Mutations in *uPAR*, the receptor for hepatocyte growth factor/scatter factor (HGF/SF), lead to aberrant migration of Parvalbumin cell precursors specifically and the adult mice display increased anxiety compared to littermates (Powell et al., 2003a). Finally, DISC1 (Disrupted-in-Schizophrenia-1) represents a factor crucial for neurite outgrowth and mutations in this gene can lead to schizophrenia in patients (Kamiya et al., 2005; Ayhan et al., 2011).

In addition to seizure-based diseases, interneuron deficiencies have been implicated in schizophrenia; whereby a reduction in GABAergic synapses by 40% have been noted in the post mortem schizophrenic prefrontal cortex (Lewis, 2000). Also, similar studies revealed reductions in most interneuron markers in developing schizophrenic brains (Fung et al., 2010). Interneurons have also been implicated in bipolar disorder, anxiety disorders and Tourette's syndrome (Jetty et al., 2001; Kalanithi et al., 2005).

## Proof-of-concept transplantation studies

One third of epileptics are resistant to current drug treatments and all antiepileptics have major side-effects (Loring et al., 2007; Loescher et al., 2008). Surgical resection of the seizure focal point can be beneficial but depends on the function of the brain location and is therefore only possible in a subset of patients. Therefore, novel therapeutic avenues are of great interest.

The potential for cell replacement therapeutic strategies for treating interneuronopathies has been demonstrated in the literature and is summarized in Table 1. The first critical evidence that promoted ventral forebrain-derived GABAergic cells was the extensive migrational capacity of grafted interneurons after cortical transplantation. Compared with LGE and cortically-derived cells that show little migration, MGE derivatives migrate several millimeters into the cortex (Wichterle et al., 1999). These grafted cells have the ability to differentiate into interneurons that express the correct markers, display characteristic physiological features and integrate to provide inhibitory synaptic activity (Alvarez-Dolado et al., 2006).

**Table 1:** **Transplantation of fetal-derived interneuron precursors**.

**Author**	**Transplanted tissue**	**Model**	**Results**
Baraban et al., 2009	E13.5 MGE	Kv1.1 mutant mouse Epilepsy model	GABAergic cells, increased GABAergic synapses. PV, SST, CR, NPY Reduced episodes of seizure and duration 30 days post-transplant
Calcagnotto et al., 2010b	E12.5 MGE tissue	SSP-Sap injected cortex	Restored inhibition within cortex
Calcagnotto et al., 2010a	MGE tissue	Maximum electroconvulsive shock model	Reduced seizure and decreased mortality PV, CR, NPY, 2 months after graft
De la Cruz et al., 2011	E13.5 MGE tissue (ventral vs. dorsal)	Ictal discharges induced by 4-AP	Attenuated propagation of ictal discharges. 2.5–8 weeks after transplant
Waldau et al., 2010	NSCs expanded from E14 MGE	Kainic acid induced epilepsy	Reduced frequency, duration and severity of seizure 3 months after graft
Zipancic et al., 2010	E12.5 MGE	SSP-Sap	Repopulate hippocampus after 2 months, Reduced seizure severity and mortality
Alvarez-Dolado et al., 2006	E12.5–E13.5		Evident interneuron morphology, expression, physiology. Increased GABAergic synaptic activity on pyramidal neurons Up to after 60 days

Details of tissue source and preparation, animal model and a summary of the results are described. Abbreviations: MGE, medial ganglionic eminence; NSC, neural stem cell; 4-AP 4-aminopyridine; PV, Parvalbumin; SST, Somatostatin; CR, calretinin; NPY, neuropeptide Y.

To investigate the potential of MGE-derived tissue to reverse the diseased state, several studies have grafted fetal GABAergic precursors into seizure model rodents. Typically MGE tissue from E12.5–E14.5 mouse embryos (a time of major interneurogenesis in the mouse embryo) is dissociated and grafted into the cortex (Xu et al., 2004). Baraban and colleagues demonstrated that wild-type MGE-tissue grafted into the cortex of a Shaker-like model of epilepsy (*Kv1.1* knockout mice) reduced the number and duration of spontaneous seizures 30 days after transplantation (Baraban et al., 2009).

The convulsive drug 4-aminopyridine (4-AP) is able to elicit focal ictal-like events due to evoked neurotransmitter release. Transplantation of E13.5 MGE cells are able to reduce the power of the seizure 2.5–8 weeks post grafting (De la Cruz et al., 2011). Interestingly, there was no relationship between graft effectiveness and the number of MGE cells transplanted in this study.

Maximum electroconvulsive shock (MES)-induced epileptic rodents have unaltered brain circuitry and so represent a good model for chronic epilepsy. Rodents that received MGE grafts in this model displayed a higher threshold to MES and a lower mortality rate 2 months after grafting (Calcagnotto et al., 2010a). Kainic acid-induced mouse models of chronic temporal lobe epilepsy have been grafted with neural stem cells (NSCs) expanded from E14 MGE tissues. Cells were grown *in vitro* using EGF and FGF2 before being transplanted into rodent hippocampus. The authors observed reduced frequency and severity of seizures three months after transplantation, although cognitive function (learning and memory) was not improved (Waldau et al., 2010).

Injection of neurotoxin-conjugated Substance P (SSP-Sap), that targets interneurons expressing the Substance P receptor NK-1, has been used to selectively degrade interneurons in the hippocampus (Martin and Sloviter, 2001). This leads to reduced numbers of Somatostatin-, NPY- and NK-1-expressing interneurons while the Calretinin and Parvalbumin subpopulations unaffected or slightly reduced, respectively. The group of Alvarez-Dolado demonstrated that E12.5 MGE-derived cells were able to repopulate the SSP-Sap treated hippocampus two months after grafting and increase inhibitory synaptic events on hippocampal pyramidal cells. This was associated with reduced mortality rate and seizure severity (Calcagnotto et al., 2010b; Zipancic et al., 2010).

Other cell sources have been shown to have potential benefit in models of interneuron disease. Firstly, NSCs derived from 15 week human fetal tissue were injected into the rat tail vein of a pilocarpine model. GAD67 expressing cells were detected in the recipient hippocampus which was accompanied by reduced pyramidal cell excitability (Chu et al., 2004). Neonatal hippocampal-derived NSCs have displayed an ability to differentiate into neurons and glia (Shetty et al., 2008). However, these cells have limited GABAergic potential, especially compared to ventral-forebrain-derived NSCs (He et al., 2001).

Other strategies tested to reverse epilepsy include transplanting cells constitutively expressing GABA to replace the inhibitory deficiency (Gernert et al., 2002; Thompson and Suchomelova, 2004; Thompson, 2005), or to inhibit the action of adenosine kinase which degrades the anticonvulsive drug adenosine in the injured brain (Huber et al., 2001; Guttinger et al., 2005; Ren et al., 2007; Li et al., 2008). These strategies showed limited benefits as the cells do not integrate into the cortical circuits and are not activity dependent; features that may be required to reduce seizures. Finally, ventral telencephalic tissue grafted into the ventral midbrain [substantia nigra (SN) pars reticulata] also demonstrated a reduction in seizure propagation (Loscher et al., 1998). This tissue has been implicated seizure propagation in various epilepsy models.

Although interneuron dysfunction has been implicated in neuropsychiatric diseased states such as anxiety disorders and attention deficit disorder, the potential use of cellular replacement therapy has not been addressed (Jetty et al., 2001; Boy et al., 2011; Edden et al., 2012).

## Interneurons from pluripotent stem cells

Human ESCs and iPSCs can be differentiated into any somatic cell type and offer an unlimited supply of medically important cells (Thomson et al., 1998). These cells may be employed to elucidate disease etiology and develop novel therapies, including putative cell replacement strategies. The goal of stem cell research is to understand lineage and cell fate specification in normal development in order to produce disease-relevant and functional post mitotic cells. Several lines of study have employed morphogenic conditions capable of directing mouse and human ESCs toward a ventral telencephalic fate. However, when compared to some other neuronal cells, such as midbrain dopamine neurons (Jaeger et al., 2011; Kriks et al., 2011), relatively little is known on how to control human pluripotent stem cell differentiation into distinct cortical interneuron subtypes (for current progress see Table 2).

**Table 2:** **Progress in ESC-derived cortical interneurons and their transplantation *in vivo***.

**Author**	**Species**	**Differentiation**	**Strategy**	**Growth factors**	***In vitro***	***In vivo***
Goulburn et al., 2011	Human	Spin EBs	NKX2.1 reporter	FGF2 and RA	Appropriate progenitor markers GABAergic neurons, PV, SST expression. Electrophysiology and migration	Migration capacity and expressed GABA
Cambray et al., 2012	Human and mouse	Adherent monolayer		Activin	CGE markers, CR neurons	Bipolar orientated cells in cortex
Maisano et al., 2012	Mouse	Adherent monolayer	Sox1 Reporter	HH agonist	CR and CB expression	TLE model, PV, CR, CB, small effect on mossy fiber sprouting, electrophysiology
Maroof et al., 2010	Mouse	EBs	Lxh6 reporter	SHH, IGF, FGF2	Interneuron precursor expression,	Migration in cortex, PV, SST Electrophysiology, 9 month survival.
Danjo et al., 2011	Mouse	Spin EBs	Foxg1 reporter	SHH, FGF8 vs. FGF15/19	Nkx2.1 expression, PV, SST, NPY, CR	Slice culture migration
Ma et al., 2012	Human	EBs		Shh	Nkx2.1, Gsx2	
Li et al., 2009	Human	EBs	Gli3 RNAi	Shh, Dkk1	Nkx2.1, Gsh2, Isl1, GAD67	
Watanabe et al., 2007	Human	EBs		Shh	Nkx2.1	
Watanabe et al., 2005	Mouse	EBs		Dkk1, LeftyA, Shh	Nkx2.1, Gsx2, Isl1	
Gaspard et al., 2008	Mouse	Adherent monolayer		Endogenous Shh in basal conditions	Nkx2.1, multipolar cells, inhibitory synaptic activity	

Details of species of ESCs used, differentiation technique and precise strategies and the results both in vivo and in vitro are given. Abbreviations: EB, embryoid body; HH, hedgehog; PV, Parvalbumin; SST, Somatostatin; CGE, caudal ganglionic eminence; CR, calretinin; CB, Calbindin; NPY, neuropeptide Y; TLE, temporal lobe epilepsy.

Ruschenschmidt et al. described the first ESC-derived neurons to be grafted into pilocarpine-treated chronically epileptic rats (Ruschenschmidt et al., 2005). Mouse ESCs were differentiated in aggregates termed embryoid bodies (EBs) in a neural albeit undirected manner. The resultant neurons, presumably of mixed identity, were transplanted into the hippocampus of epileptic rats. The grafts exhibited neuronal properties but did not produce any functional recovery and there was no extensive migration into the host brain, features that might be expected with interneuron-rich grafts (Wichterle et al., 1999).

Subsequent studies attempted a more directed differentiation scheme, i.e., toward a telencephalic progenitor fate. These studies demonstrate that, in serum-free conditions, neural precursors with a rostral identity can be generated from both human and mouse ESCs using an EB differentiation method (Watanabe et al., 2005, 2007). Telencephalic progenitors can also be generated using adherent monolayer-based differentiation protocols together with SMAD inhibitors in a manner parallel to the Anterior Neuroectoderm Ridge instructed development *in vivo* (Ying et al., 2003; Chambers et al., 2009).

Dorsoventral patterning of ESC-derived forebrain precursors by morphogenic gradients appears to mimic that observed during normal development. Shh is a strong ventralizing factor throughout the neural tube (Ericson et al., 1995) and specifies MGE identity through induction of *Nkx2.1*. Studies that applied graded dosage of Shh to telencephalic progenitor cells showed that a high concentration of Shh (or Shh agonists) is able to induce *Nkx2.1* and results in characteristic interneuron marker expression in ESC-derived neural cultures (Watanabe et al., 2005, 2007; Gaspard et al., 2008; Li et al., 2009; Danjo et al., 2011; Ma et al., 2012). All these studies demonstrated the instructive capabilities of Shh to promote MGE-like identity at high dose and an LGE-like fate at lower doses. Cortical fate was induced when Shh signaling was antagonized. It should be noted that *Nkx2.1* is also expressed in the ventral diencephalon and so is not specific to MGE-derived interneuron progenitors (Ohyama et al., 2005).

A number of studies have investigated the functionality of *in vitro* derived interneurons and performed grafting studies of ESC-derived cultures into rodent model organisms. Maroof et al. utilized an EB based differentiation scheme followed by Shh treatment to produce ventral telencephalic-like cells from mouse ESCs (Maroof et al., 2010). Using an Lhx6-reporter that specifically marks the migrating MGE-derivatives, the authors purified putative interneurons by FACS and grafted the GFP expressing cells into the adult mouse cortex. They observed extensive cellular migration and survival. Graft-derived cells exhibited classical interneuron marker expression (predominantly Parvalbumin and Somatostatin) and electrophysiological properties nine months post-transplantation.

Maisano et al. subsequently produced interneuron-like cells from mouse ESCs via adherent monolayer differentiation (Maisano et al., 2012). To promote ventral telencephalic differentiation, the authors used a Shh agonist and witnessed faithful differentiation *in vitro*. Following transplantation into the hippocampus of a pilocarpine-based mouse model for temporal lobe epilepsy, the neural precursors reliably formed GABAergic neurons, replacing those lost in this model. These cells were physiologically indicative of GABAergic cells and expressed Parvalbumin, Calbindin, and Calretinin but not Somatostatin. This may be explained by the sensitivity of Somatostatin-expressing cells to pilocarpine treatment. Unfortunately, the authors did not observe a statistical reduction in mossy fiber sprouting, a characteristic trait of epilepsy.

Goulburn et al. utilized an NKX2.1-GFP human ESC reporter cell line to purify ventral telencephalic-like cells (Goulburn et al., 2011). The group used an EB-based neural induction protocol with FGF2 and retinoid acid (RA) treatment to promote NKX2.1 promoter-driven GFP expression. Although the use of RA is contentious, the RA signaling pathway has been shown to be relevant for early forebrain development (Schneider et al., 2001; Stavridis et al., 2010; Chatzi et al., 2011). Sorted NKX2.1-GFP^+^ cells gave rise to GABAergic and Somatostatin-expressing neurons *in vitro* that displayed migration capacity both *in vitro* and *in vivo*. These sorted cells also expressed diencephalic marker genes; a second region that is dependent on NKX2.1 expression. Therefore, all the evidence thus far indicates that the MGE is patterned by Shh and that Shh can induce ESCs to generate at least some subtypes of MGE-derived interneurons.

A recent study described the generation of Calretinin-expressing cortical interneurons from mouse and human ESCs/iPSCs (Cambray et al., 2012). During development, the majority of Calretinin interneurons are believed to be generated in the dorsal CGE, which appears to be non-responsive to Shh [see Figure 1 (Xu et al., 2010)]. Using an adherent monolayer differentiation strategy, Cambray et al. reported that signaling via Activin induces the expression of genes that are associated with the CGE and promote the generation of Calretinin-expressing interneurons with minimal generation of MGE-derived interneuron subtypes (Cambray et al., 2012). Following transplantation, these neurons exhibited bipolar morphology in the adult rodent cortex. Members of fibroblast growth factors (FGFs) have also been reported to specify either an MGE or CGE-like fate (FGF8 vs. FGF15/19 respectively) as well as their neuronal derivatives (Danjo et al., 2011).

Together these studies provide a strong argument for the potential benefit of investigating further ESC-derived interneurons for the treatment of interneuronopathies. It is clear that the vast intricacies of cell specification require a great deal of work to improve our understanding of the developmental events, for example genome-wide investigations. As well as the patterning of interneuron precursors, aspects such as developmental timing and transplantation site may have a huge impact on the final phenotype of a transplanted cell through exogenous signaling environments. The safety of ESC-derived cells must be carefully considered before transplantation; namely proliferation of undifferentiated cells, side-effects of cell heterogeneity and complications associated with surgery. These factors will need to be investigated fully to maximize the potential of this technology.

## Future perspectives

The power of ESCs in generating subpallial-derived interneurons is starting to be unlocked. Future work is needed to expand the repertoire of interneuron subtypes generated from hESCs and iPSCs. Precise temporal control of developmental signaling pathways may be critical to direct preferential production of individual interneuron subtypes. For example, within the MGE, progenitors respond to higher doses of Shh preferentially to give rise to Somatostatin neurons while the progenitors that experience lower level of Shh signaling differentiate into Parvalbumin-expressing neurons (Wonders et al., 2008; Xu et al., 2010; Inan et al., 2012). It would be interesting to see how these developmental findings translate to neuronal subtype specification in stem cell systems. The development of novel interneuron differentiation protocols would benefit from better characterization of interneuron diversity in model organisms that establish links between cellular morphology, neurochemical phenotypes, synaptic connection types, and electrophysiological properties of the interneurons.

Neuropsychiatric disorders present a major burden to modern society. The major limitation for the development of new treatments has been the lack of defined etiology and limited knowledge of the disease pathophysiology (Lewis and Gonzalez-Burgos, 2006). Studies of these diseases rely on imaging techniques and post-mortem analysis of patients' brains. It is generally recognized that neuropsychiatric diseases are developmental disorders due to aberrant neuronal wiring. In this regard, human ESCs provide a robust *in vitro* model to gain insight into human cortical development. Much of our knowledge on the human brain and psychiatric diseases stems from rodent models. However, the human cortex is significantly larger than the rodent's with extra cortical germinal layers (Molnar et al., 2011). Particularly relevant to interneuron disease is the fact that the human and rodent brains differ in the ratio of inhibitory/excitatory neurons. This difference may impact on interneuron regulated network function and hence the extent to which rodent models can mimic human conditions. Furthermore, the genetic variants identified in psychiatric patients often involve long range genomic duplications or deletions spanning multiple genes. Such complex genetic variants can be difficult to model in an animal model. Patient-derived iPSCs offer unprecedented opportunity to generate human neurons with identical genetic information to that of patients to unravel the cellular defects and underlying molecular mechanisms caused by disease gene mutations. Therefore, hiPSCs offers a tractable alternative to rodent models.

The success of fetal tissue-based grafts has paved the way for stem cell-based interneuron replacement strategies. However, stem cell-derived interneurons have so far not been shown to produce a functional effect on disease models (Maroof et al., 2010; Goulburn et al., 2011; Cambray et al., 2012; Maisano et al., 2012). Furthermore, certain hurdles should be kept in mind with regard to interneuron transplantation therapy. Firstly, a proportion of pharmacoresistant epileptic patients do not respond to exogenous GABA administration as a treatment. Would grafted GABAergic cells have a beneficial effect in this paradigm? Cortical interneurons also have much interneuron-to-interneuron synaptic contact. For this reason, the subtype of grafted neurons must be carefully monitored to prevent altered activity of existing inhibitory cells within the disease setting. As discussed, the developmental timing and the transplantation site may have extrinsic effects on a transplanted cell's fate and so must be appreciated prior to transplant optimization. Finally, as with all stem cell-derived cell therapy postulations, a number of caveats may limit the safety of such an approach. For example, contaminating undifferentiated cells from the *in vitro* differentiation process may lead to cellular overgrowths and teratomas as well as other, off-target cells leading to adverse effects.

Nevertheless, the field of cortical interneuron dysfunction is set to welcome ESC based strategies that will enable a big step toward disease modeling and cell-based therapies for treating common neurological disorders.

### Conflict of interest statement

The authors declare that the research was conducted in the absence of any commercial or financial relationships that could be construed as a potential conflict of interest.

## References

[B1] Akbarian S. Huang H. S. (2006). Molecular and cellular mechanisms of altered GAD1/GAD67 expression in schizophrenia and related disorders. Brain Res. Rev. 52, 293–304 10.1016/j.brainresrev.2006.04.001 16759710

[B2] Alvarez-Dolado M. Calcagnotto M. E. Karkar K. M. Southwell D. G. Jones-Davis D. M. Estrada R. C. (2006). Cortical inhibition modified by embryonic neural precursors grafted into the postnatal brain. J. Neurosci. 26, 7380–7389 10.1523/JNEUROSCI.1540-06.2006 16837585 PMC1550786

[B3] Anderson S. A. Eisenstat D. D. Shi L. Rubenstein J. L. R. (1997). Interneuron migration from basal forebrain to neocortex: dependence on Dlx genes. Science 278, 474–476 10.1126/science.278.5337.474 9334308

[B4] Ascoli G. A. Alonso-Nanclares L. Anderson S. A. Barrionuevo G. Benavides-Piccione R. Burkhalter A. (2008). Petilla terminology: nomenclature of features of GABAergic interneurons of the cerebral cortex. Nat. Rev. Neurosci. 9, 557–568 10.1038/nrn2402 18568015 PMC2868386

[B5] Ayhan Y. Abazyan B. Nomura J. Kim R. Ladenheim B. Krasnova I. N. (2011). Differential effects of prenatal and postnatal expressions of mutant human DISC1 on neurobehavioral phenotypes in transgenic mice: evidence for neurodevelopmental origin of major psychiatric disorders. Mol. Psychiatry 16, 293–306 10.1038/mp.2009.144 20048751 PMC2914807

[B6] Baraban S. C. Southwell D. G. Estrada R. C. Jones D. L. Sebe J. Y. Alfaro-Cervello C. (2009). Reduction of seizures by transplantation of cortical GABAergic interneuron precursors into Kv1.1 mutant mice. Proc. Natl. Acad. Sci. U.S.A. 106, 15472–15477 10.1073/pnas.0900141106 19706400 PMC2741275

[B7] Batista-Brito R. Fishell G. (2009). The developmental integration of cortical interneurons into a functional network. Curr. Top. Dev. Biol. 87, 81–118 10.1016/S0070-2153(09)01203-4 19427517 PMC4465088

[B8] Boy F. Evans C. J. Edden R. A. E. Lawrence A. D. Singh K. D. Husain M. (2011). Dorsolateral prefrontal gamma-aminobutyric acid in men predicts individual differences in rash impulsivity. Biol. Psychiatry 70, 866–872 10.1016/j.biopsych.2011.05.030 21757187 PMC3192031

[B9] Braz J. M. Sharif-Naeini R. Vogt D. Kriegstein A. Alvarez-Buylla A. Rubenstein J. L. (2012). Forebrain GABAergic neuron precursors integrate into adult spinal cord and reduce injury-induced neuropathic pain. Neuron 74, 663–675 10.1016/j.neuron.2012.02.033 22632725 PMC3361692

[B10] Butt S. J. B. Fuccillo M. Nery S. Noctor S. Kriegstein A. Corbin J. G. (2005). The temporal and spatial origins of cortical interneurons predict their physiological subtype. Neuron 48, 591–604 10.1016/j.neuron.2005.09.034 16301176

[B11] Calcagnotto M. E. Ruiz L. P. Blanco M. M. Santos-Junior J. G. Valente M. F. Patti C. (2010a). Effect of neuronal precursor cells derived from medial ganglionic eminence in an acute epileptic seizure model. Epilepsia 51 Suppl. 3, 71–75 10.1111/j.1528-1167.2010.02614.x 20618405

[B12] Calcagnotto M. E. Zipancic I. Piquer-Gil M. Mello L. E. Alvarez-Dolado M. (2010b). Grafting of GABAergic precursors rescues deficits in hippocampal inhibition. Epilepsia 51, 591–604 10.1111/j.1528-1167.2010.02613.x 20618404

[B13] Cambray S. Arber C. Little G. Dougalis A. G. de Paola V. Ungless M. A. (2012). Activin induces cortical interneuron identity and differentiation in embryonic stem cell-derived telencephalic neural precursors. Nat. Commun. 3:841 10.1038/ncomms1817 22588303

[B14] Chambers S. M. Fasano C. A. Papapetrou E. P. Tomishima M. Sadelain M. Studer L. (2009). Highly efficient neural conversion of human ES and iPS cells by dual inhibition of SMAD signaling. Nat. Biotechnol. 27, 275–280 10.1038/nbt.1529 19252484 PMC2756723

[B15] Chatzi C. Brade T. Duester G. (2011). Retinoic acid functions as a key GABAergic differentiation signal in the basal ganglia. PLoS Biol. 9:e1000609 10.1371/journal.pbio.1000609 21532733 PMC3075211

[B16] Chiang C. Ying L. T. T. Lee E. Young K. E. Corden J. L. Westphal H. (1996). Cyclopia and defective axial patterning in mice lacking Sonic hedgehog gene function. Nature 383, 407–413 10.1038/383407a0 8837770

[B17] Chu K. Kim M. Jung K. H. Jeon D. Lee S. T. Kim J. (2004). Human neural stem cell transplantation reduces spontaneous recurrent seizures following pilocarpine-induced status epilepticus in adult rats. Brain Res. 1023, 213–221 10.1016/j.brainres.2004.07.045 15374747

[B18] Cossart R. Dinocourt C. Hirsch J. C. Merchan-Perez A. De Felipe J. Ben-Ari Y. (2001). Dendritic but not somatic GABAergic inhibition is decreased in experimental epilepsy. Nat. Neurosci. 4, 52–62 10.1038/82900 11135645

[B19] Danjo T. Eiraku M. Muguruma K. Watanabe K. Kawada M. Yanagawa Y. (2011). Subregional specification of embryonic stem cell-derived ventral telencephalic tissues by timed and combinatory treatment with extrinsic signals. J. Neurosci. 31, 1919–1933 10.1523/JNEUROSCI.5128-10.2011 21289201 PMC6623725

[B20] DeFelipe J. (1997). Types of neurons, synaptic connections and chemical characteristics of cells immunoreactive for calbindin-D28K, parvalbumin and calretinin in the neocortex. J. Chem. Neuroanat. 14, 1–19 10.1016/S0891-0618(97)10013-8 9498163

[B21] DeFelipe J. (2002). Cortical interneurons: from Cajal to 2001. Prog. Brain Res. 136, 215–238 12143384 10.1016/s0079-6123(02)36019-9

[B22] De la Cruz E. Zhao M. Guo L. Ma H. Anderson S. A. Schwartz T. H. (2011). Interneuron Progenitors attenuate the power of acute focal ictal discharges. Neurotherapeutics 8, 763–773 10.1007/s13311-011-0058-9 21748528 PMC3250298

[B23] Du T. Xu Q. Ocbina P. J. Anderson S. A. (2008). NKX2.1 specifies cortical interneuron fate by activating Lhx6. Development 135, 1559–1567 10.1242/dev.015123 18339674

[B24] Edden R. A. E. Crocetti D. Zhu H. Gilbert D. L. Mostofsky S. H. (2012). Reduced GABA concentration in attention-deficit/hyperactivity disorder. Arch. Gen. Psychiatry 69, 750–753 10.1001/archgenpsychiatry.2011.2280 22752239 PMC3970207

[B25] Ericson J. Muhr J. Placzek M. Lints T. Jessell T. M. Edlund T. (1995). Sonic hedgehog induces the differentiation of ventral forebrain neurons - a common signal for ventral patterning within the neural-tube. Cell 81, 747–756 10.1016/0092-8674(95)90536-7 7774016

[B26] Faux C. Rakic S. Andrews W. Britto J. M. (2012). Neurons on the move: migration and lamination of cortical interneurons. Neurosignals 20, 168–189 10.1159/000334489 22572780

[B27] Fertuzinhos S. Krsnik Z. Kawasawa Y. I. Rasin M. R. Kwan K. Y. Chen J. G. (2009). Selective depletion of molecularly defined cortical interneurons in human holoprosencephaly with severe striatal hypoplasia. Cereb. Cortex 19, 2196–2207 10.1093/cercor/bhp009 19234067 PMC2722430

[B28] Flames N. Pla R. Gelman D. M. Rubenstein J. L. R. Puelles L. Marin O. (2007). Delineation of multiple subpallial progenitor domains by the combinatorial expression of transcriptional codes. J. Neurosci. 27, 9682–9695 10.1523/JNEUROSCI.2750-07.2007 17804629 PMC4916652

[B29] Friocourt G. Parnavelas J. G. (2010). Mutations in ARX result in several defects involving GABAergic neurons. Front. Cell. Neurosci. 4:4 10.3389/fncel.2010.00004 20300201 PMC2841486

[B30] Fung S. J. Webster M. J. Sivagnanasundaram S. Duncan C. Elashoff M. Weickert C. S. (2010). Expression of interneuron markers in the dorsolateral prefrontal cortex of the developing human and in schizophrenia. Am. J. Psychiatry 167, 1479–1488 10.1176/appi.ajp.2010.09060784 21041246

[B31] Gaspard N. Bouschet T. Hourez R. Dimidschstein J. Naeije G. van den Ameele J. (2008). An intrinsic mechanism of corticogenesis from embryonic stem cells. Nature 455, 351–357 10.1038/nature07287 18716623

[B32] Gernert M. Thompson K. W. Loscher W. Tobin A. J. (2002). Genetically engineered GABA-producing cells demonstrate anticonvulsant effects and long-term transgene expression when transplanted into the central piriform cortex of rats. Exp. Neurol. 176, 183–192 10.1006/exnr.2002.7914 12093095

[B33] Gouder N. Scheurer L. Fritschy J. M. Boison D. (2004). Overexpression of adenosine kinase in epileptic hippocampus contributes to epileptogenesis. J. Neurosci. 24, 692–701 10.1523/JNEUROSCI.4781-03.2004 14736855 PMC6729249

[B34] Goulburn A. L. Alden D. Davis R. P. Micallef S. J. Ng E. S. Yu Q. C. (2011). A targeted NKX2.1 human embryonic stem cell reporter line enables identification of human basal forebrain derivatives. Stem Cells 29, 462–473 10.1002/stem.587 21425409

[B35] Guttinger M. Fedele D. Koch P. Padrun V. Pralong W. F. Brustle O. (2005). Suppression of kindled seizures by paracrine adenosine release from stem cell-derived brain implants. Epilepsia 46, 1162–1169 10.1111/j.1528-1167.2005.61804.x 16060924

[B36] Haider B. Duque A. Hasenstaub A. R. McCormick D. A. (2006). Neocortical network activity *in vivo* is generated through a dynamic balance of excitation and inhibition. J. Neurosci. 26, 4535–4545 10.1523/JNEUROSCI.5297-05.2006 16641233 PMC6674060

[B37] He W. L. Ingraham C. Rising L. Goderie S. Temple S. (2001). Multipotent stem cells from the mouse basal forebrain contribute GABAergic neurons and oligodendrocytes to the cerebral cortex during embryogenesis. J. Neurosci. 21, 8854–8862 11698597 10.1523/JNEUROSCI.21-22-08854.2001 PMC6762260

[B38] Hensch T. K. (2005). Critical period plasticity in local cortical circuits. Nat. Rev. Neurosci. 6, 877–888 10.1038/nrn1787 16261181

[B39] Huber A. Padrun V. Deglon N. Aebischer P. Mohler H. Boison D. (2001). Grafts of adenosine-releasing cells suppress seizures in kindling epilepsy. Proc. Natl. Acad. Sci. U.S.A. 98, 7611–7616 10.1073/pnas.131102898 11404469 PMC34716

[B40] Hussman J. P. (2001). Suppressed GABAergic inhibition as a common factor in suspected etiologies of autism. J. Autism Dev. Disord. 31, 247–248 11450824 10.1023/a:1010715619091

[B41] Inan M. Welagen J. Anderson S. A. (2012). Spatial and temporal bias in the mitotic origins of somatostatin- and parvalbumin-expressing interneuron subgroups and the chandelier subtype in the medial ganglionic eminence. Cereb. Cortex 22, 820–827 10.1093/cercor/bhr148 21693785 PMC3450921

[B42] Jaeger I. Arber C. Risner-Janiczek J. R. Kuechler J. Pritzsche D. Chen I. C. (2011). Temporally controlled modulation of FGF/ERK signaling directs midbrain dopaminergic neural progenitor fate in mouse and human pluripotent stem cells. Development 138, 4363–4374 10.1242/dev.066746 21880784 PMC3177308

[B43] Jakovcevski I. Mayer N. Zecevic N. (2011). Multiple origins of human neocortical interneurons are supported by distinct expression of transcription factors. Cereb. Cortex 21, 1771–1782 10.1093/cercor/bhq245 21139075 PMC3138511

[B44] Jetty P. V. Charney D. S. Goddard A. W. (2001). Neurobiology of generalized anxiety disorder. Psychiatr. Clin. N. Am. 24, 1771–1782 11225510 10.1016/s0193-953x(05)70207-0

[B45] Kalanithi P. S. A. Zheng W. Kataoka Y. DiFiglia M. Grantz H. Saper C. B. (2005). Altered parvalbumin-positive neuron distribution in basal ganglia of individuals with Tourette syndrome. Proc. Natl. Acad. Sci. U.S.A. 102, 13307–13312 10.1073/pnas.0502624102 16131542 PMC1201574

[B46] Kamiya A. Kubo K. Tomoda T. Takaki M. Youn R. Ozeki Y. (2005). A schizophrenia-associated mutation of DISC1 perturbs cerebral cortex development. Nat. Cell Biol. 7, 1167–1178 10.1038/ncb1328 16299498

[B47] Kawaguchi Y. Kubota Y. (1997). GABAergic cell subtypes and their synaptic connections in rat frontal cortex. Cereb. Cortex 7, 476–486 10.1093/cercor/7.6.476 9276173

[B48] Klausberger T. Somogyi P. (2008). Neuronal diversity and temporal dynamics: the unity of hippocampal circuit operations. Science 321, 53–57 10.1126/science.1149381 18599766 PMC4487503

[B49] Kriks S. Shim J. W. Piao J. H. Ganat Y. M. Wakeman D. R. Xie Z. (2011). Dopamine neurons derived from human ES cells efficiently engraft in animal models of Parkinson's disease. Nature 480, 547–551 10.1038/nature10648 22056989 PMC3245796

[B50] Kubota Y. Hattori R. Yui Y. (1994). Three distinct subpopulations of GABAergic neurons in rat frontal agranular cortex. Brain Res. 649, 159–173 10.1016/0006-8993(94)91060-X 7525007

[B51] Lavdas A. A. Grigoriou M. Pachnis V. Parnavelas J. G. (1999). The medial ganglionic eminence gives rise to a population of early neurons in the developing cerebral cortex. J. Neurosci. 19, 7881–7888 10479690 10.1523/JNEUROSCI.19-18-07881.1999 PMC6782477

[B52] Letinic K. Zoncu R. Rakic P. (2002). Origin of GABAergic neurons in the human neocortex. Nature 417, 645–649 10.1038/nature00779 12050665

[B53] Lewis D. A. (2000). GABAergic local circuit neurons and prefrontal cortical dysfunction in schizophrenia. Brain Res. Rev. 31, 270–276 10.1016/S0165-0173(99)00042-9 10719153

[B54] Lewis D. A. Gonzalez-Burgos G. (2006). Pathophysiologically based treatment interventions in schizophrenia. Nat. Med. 12, 1016–1022 10.1038/nm1478 16960576

[B55] Lewis D. A. Hashimoto T. Volk D. W. (2005). Cortical inhibitory neurons and schizophrenia. Nat. Rev. Neurosci. 6, 312–324 10.1038/nrn1648 15803162

[B56] Li T. F. Ren G. Y. Lusardi T. Wilz A. Lan J. Q. Iwasato T. (2008). Adenosine kinase is a target for the prediction and prevention of epileptogenesis in mice. J. Clin. Investig. 118, 571–582 10.1172/JCI33737 18172552 PMC2157568

[B57] Li X.-J. Zhang X. Johnson M. A. Wang Z.-B. LaVaute T. Zhang S.-C. (2009). Coordination of sonic hedgehog and Wnt signaling determines ventral and dorsal telencephalic neuron types from human embryonic stem cells. Development 136, 4055–4063 10.1242/dev.036624 19906872 PMC2778748

[B58] Loescher W. Gernert M. Heinemann U. (2008). Cell and gene therapies in epilepsy - promising avenues or blind alleys? Trends Neurosci. 31, 62–73 10.1016/j.tins.2007.11.012 18201772

[B59] Long J. E. Cobos I. Potter G. B. Rubenstein J. L. R. (2009). Dlx1and2 and Mash1 transcription factors control MGE and CGE patterning and differentiation through parallel and overlapping pathways. Cereb. Cortex 19 Suppl. 1, i96–i106 10.1093/cercor/bhp045 19386638 PMC2693539

[B60] Loring D. W. Marino S. Meador K. J. (2007). Neuropsychological and behavioral effects of antiepilepsy drugs. Neuropsychol. Rev. 17, 413–425 10.1007/s11065-007-9043-9 17943448

[B61] Loscher W. Ebert U. Lehmann H. Rosenthal C. Nikkhah G. (1998). Seizure suppression in kindling epilepsy by grafts of fetal GABAergic neurons in rat substantia nigra. J. Neurosci. Res. 51, 196–209 10.1002/(SICI)1097-4547(19980115)51:2<196::AID-JNR8>3.0.CO;2-8 9469573

[B62] Ma L. Hu B. Liu Y. Vermilyea S. C. Liu H. Gao L. (2012). Human embryonic stem cell-derived GABA neurons correct locomotion deficits in quinolinic acid-lesioned mice. Cell Stem Cell 10, 455–464 10.1016/j.stem.2012.01.021 22424902 PMC3322292

[B63] Maisano X. Carpentino J. Becker S. Lanza R. Aaron G. Grabel L. (2009). Embryonic stem cell-derived neural precursor grafts for treatment of temporal lobe epilepsy. Neurotherapeutics 6, 263–277 10.1016/j.nurt.2009.01.011 19332319 PMC2830617

[B64] Maisano X. Litvina E. Tagliatela S. Aaron G. B. Grabel L. B. Naegele J. R. (2012). Differentiation and functional incorporation of embryonic stem cell-derived GABAergic interneurons in the dentate gyrus of mice with temporal lobe epilepsy. J. Neurosci. 32, 46–61 10.1523/JNEUROSCI.2683-11.2012 22219269 PMC3548598

[B65] Markram H. Toledo-Rodriguez M. Wang Y. Gupta A. Silberberg G. Wu C. Z. (2004). Interneurons of the neocortical inhibitory system. Nat. Rev. Neurosci. 5, 793–807 10.1038/nrn1519 15378039

[B66] Maroof A. M. Brown K. Shi S.-H. Studer L. Anderson S. A. (2010). Prospective isolation of cortical interneuron precursors from mouse embryonic stem cells. J. Neurosci. 30, 4667–4675 10.1523/JNEUROSCI.4255-09.2010 20357117 PMC2868507

[B67] Martin J. L. Sloviter R. S. (2001). Focal inhibitory interneuron loss and principal cell hyperexcitability in the rat hippocampus after microinjection of a neurotoxic conjugate of saporin and a peptidase-resistant analog of Substance, P. J. Comp. Neurol. 436, 127–152 11438920

[B68] Martinez-Cerdeno V. Noctor S. C. Espinosa A. Ariza J. Parker P. Orasji S. (2010). Embryonic MGE precursor cells grafted into adult rat striatum integrate and ameliorate motor symptoms in 6-OHDA-lesioned rats. Cell Stem Cell 6, 238–250 10.1016/j.stem.2010.01.004 20207227 PMC4075336

[B69] Miyoshi G. Fishell G. (2011). GABAergic interneuron lineages selectively sort into specific cortical layers during early postnatal development. Cereb. Cortex 21, 845–852 10.1093/cercor/bhq155 20732898 PMC3059886

[B70] Molnar Z. Vasistha N. A. Garcia-Moreno F. (2011). Hanging by the tail: progenitor populations proliferate. Nat. Neurosci. 14, 538–540 10.1038/nn.2817 21522143

[B71] Nadarajah B. Alifragis P. Wong R. O. L. Parnavelas J. G. (2002). Ventricle-directed migration in the developing cerebral cortex. Nat. Neurosci. 5, 218–224 10.1038/nn813 11850632

[B72] Nery S. Fishell G. Corbin J. G. (2002). The caudal ganglionic eminence is a source of distinct cortical and subcortical cell populations. Nat. Neurosci. 5, 1279–1287 10.1038/nn971 12411960

[B73] Ohyama K. Ellis P. Kimura S. Placzek M. (2005). Directed differentiation of neural cells to hypothalamic dopaminergic neurons. Development 132, 5185–5197 10.1242/dev.02094 16284116

[B74] Parra P. Gulyas A. I. Miles R. (1998). How many subtypes of inhibitory cells in the hippocampus? Neuron 20, 983–993 10.1016/S0896-6273(00)80479-1 9620702

[B75] Petanjek Z. Berger B. Esclapez M. (2009). Origins of cortical GABAergic neurons in the cynomolgus monkey. Cereb. Cortex 19, 249–262 10.1093/cercor/bhn078 18477686 PMC2638783

[B75a] Powell E. M. Muhlfriedel M. Bolz J. Levitt P. (2003a). Differential regulation of thalamic and cortical axonal growth by hepatocyte growth factor/scatter factor. Dev. Neurosci. 25, 197–206 10.1159/000072268 12966217

[B76] Powell E. M. Campbell D. B. Stanwood G. D. Davis C. Noebels J. L. Levitt P. (2003b). Genetic disruption of cortical interneuron development causes region- and GABA cell type-specific deficits, epilepsy, and behavioral dysfunction. J. Neurosci. 23, 622–631 12533622 10.1523/JNEUROSCI.23-02-00622.2003 PMC6741866

[B77] Rakic S. Zecevic N. (2003). Early oligodendrocyte progenitor cells in the human fetal telencephalon. Glia 41, 117–127 10.1002/glia.10140 12509802

[B78] Ren G. Li T. Lan J. Q. Wilz A. Simon R. P. Boison D. (2007). Lentiviral RNAi-induced downregulation of adenosine kinase in human mesenchymal stem cell grafts: a novel perspective for seizure control. Exp. Neurol. 208, 26–37 10.1016/j.expneurol.2007.07.016 17716659 PMC2205528

[B79] Rossignol E. (2011). Genetics and function of neocortical GABAergic interneurons in neurodevelopmental disorders. Neural Plasticity 2011:649325 10.1155/2011/649325 21876820 PMC3159129

[B80] Ruschenschmidt C. Koch P. G. Brustle O. Beck H. (2005). Functional properties of ES cell-derived neurons engrafted into the hippocampus of adult normal and chronically epileptic rats. Epilepsia 46 Suppl. 5, 174–183 10.1111/j.1528-1167.2005.01028.x 15987274

[B81] Schneider R. A. Hu D. Rubenstein J. L. R. Maden M. Helms J. A. (2001). Local retinoid signaling coordinates forebrain and facial morphogenesis by maintaining FGF8 and SHH. Development 128, 2755–2767 11526081 10.1242/dev.128.14.2755

[B82] Shetty A. K. Rao M. S. Hattiangady B. (2008). Behavior of hippocampal stem/progenitor cells following grafting into the injured aged hippocampus. J. Neurosci. Res. 86, 3062–3074 10.1002/jnr.21764 18618674 PMC2575032

[B83] Sotelo C. (2003). Viewing the brain through the master hand of Ramon y Cajal. Nat. Rev. Neurosci. 4, 71–77 10.1038/nrn1010 12511863

[B84] Stavridis M. P. Collins B. J. Storey K. G. (2010). Retinoic acid orchestrates fibroblast growth factor signalling to drive embryonic stem cell differentiation. Development 137, 881–890 10.1242/dev.043117 20179094 PMC2834455

[B85] Storm E. E. Garel S. Borello U. Hebert J. M. Martinez S. McConnell S. K. (2006). Dose-dependent functions of Fgf8 in regulating telencephalic patterning centers. Development 133, 1831–1844 10.1242/dev.02324 16613831

[B86] Sussel L. Marin O. Kimura S. Rubenstein J. L. R. (1999). Loss of Nkx2.1 homeobox gene function results in a ventral to dorsal molecular respecification within the basal telencephalon: evidence for a transformation of the pallidum into the striatum. Development 126, 3359–3370 10393115 10.1242/dev.126.15.3359

[B87] Tamamaki N. Tomioka R. (2010). Long-range GABAergic connections distributed throughout the neocortex and their possible function. Front. Neurosci. 4:202 10.3389/fnins.2010.00202 21151790 PMC3000116

[B88] Thompson K. W. (2005). Genetically engineered cells with regulatable GABA production can affect afterdischarges and behavioral seizures after transplantation into the dentate gyrus. Neuroscience 133, 1029–1037 10.1016/j.neuroscience.2005.03.003 15927406

[B89] Thompson K. W. Suchomelova L. M. (2004). Transplants of cells engineered to produce GABA suppress spontaneous seizures. Epilepsia 45, 4–12 10.1111/j.0013-9580.2004.29503.x 14692901

[B90] Thomson J. A. Itskovitz-Eldor J. Shapiro S. S. Waknitz M. A. Swiergiel J. J. Marshall V. S. (1998). Embryonic stem cell lines derived from human blastocysts. Science 282, 1145–1147 10.1126/science.282.5391.1145 9804556

[B91] Toledo-Rodriguez M. Blumenfeld B. Wu C. Z. Luo J. Y. Attali B. Goodman P. (2004). Correlation maps allow neuronal electrical properties to be predicted from single-cell gene expression profiles in rat neocortex. Cereb. Cortex 14, 1310–1327 10.1093/cercor/bhh092 15192011

[B92] Waclaw R. R. Wang B. Pei Z. L. Ehrman L. A. Campbell K. (2009). Distinct temporal requirements for the homeobox gene Gsx2 in specifying striatal and olfactory bulb neuronal fates. Neuron 63, 451–465 10.1016/j.neuron.2009.07.015 19709628 PMC2772064

[B93] Waldau B. Hattiangady B. Kuruba R. Shetty A. K. (2010). Medial ganglionic eminence-derived neural stem cell grafts ease spontaneous seizures and restore GDNF expression in a rat model of chronic temporal lobe epilepsy. Stem Cells 28, 1153–1164 10.1002/stem.446 20506409 PMC2933789

[B94] Wang X. J. Tegner J. Constantinidis C. Goldman-Rakic P. S. (2004). Division of labor among distinct subtypes of inhibitory neurons in a cortical microcircuit of working memory. Proc. Natl. Acad. Sci. U.S.A. 101, 1368–1373 10.1073/pnas.0305337101 14742867 PMC337059

[B95] Watanabe K. Kamiya D. Nishiyama A. Katayama T. Nozaki S. Kawasaki H. (2005). Directed differentiation of telencephalic precursors from embryonic stem cells. Nat. Neurosci. 8, 288–296 10.1038/nn1402 15696161

[B96] Watanabe K. Ueno M. Kamiya D. Nishiyama A. Matsumura M. Wataya T. (2007). A ROCK inhibitor permits survival of dissociated human embryonic stem cells. Nat. Biotechnol. 25, 681–686 10.1038/nbt1310 17529971

[B97] Whittington M. A. Traub R. D. (2003). Interneuron diversity series: inhibitory interneurons and network oscillations *in vitro*. Trends Neurosci. 26, 676–682 10.1016/j.tins.2003.09.016 14624852

[B98] Wichterle H. Garcia-Verdugo J. M. Herrera D. G. Alvarez-Buylla A. (1999). Young neurons from medial ganglionic eminence disperse in adult and embryonic brain. Nat. Neurosci. 2, 461–466 10.1038/8131 10321251

[B99] Willi-Monnerat S. Migliavacca E. Surdez D. Delorenzi M. Luthi-Carter R. Terskikh A. V. (2008). Comprehensive spatiotemporal transcriptomic analyses of the ganglionic eminences demonstrate the uniqueness of its caudal subdivision. Mol. Cell. Neurosci. 37, 845–856 10.1016/j.mcn.2008.01.009 18316204

[B100] Wonders C. P. Taylor L. Welagen J. Mbata I. C. Xiang J. Z. Anderson S. A. (2008). A spatial bias for the origins of intemeuron subgroups within the medial ganglionic eminence. Dev. Biol. 314, 127–136 10.1016/j.ydbio.2007.11.018 18155689 PMC2727678

[B101] Xu Q. Cobos I. De la Cruz E. Rubenstein J. L. Anderson S. A. (2004). Origins of cortical interneuron subtypes. J. Neurosci. 24, 2612–2622 10.1523/JNEUROSCI.5667-03.2004 15028753 PMC6729522

[B102] Xu Q. Guo L. H. Moore H. Waclaw R. R. Campbell K. Anderson S. A. (2010). Sonic hedgehog signaling confers ventral telencephalic progenitors with distinct cortical interneuron fates. Neuron 65, 328–340 10.1016/j.neuron.2010.01.004 20159447 PMC2868511

[B103] Ying Q. L. Stavridis M. Griffiths D. Li M. Smith A. (2003). Conversion of embryonic stem cells into neuroectodermal precursors in adherent monoculture. Nat. Biotechnol. 21, 183–186 10.1038/nbt780 12524553

[B104] Yizhar O. Fenno L. E. Prigge M. Schneider F. Davidson T. J. O'Shea D. J. (2011). Neocortical excitation/inhibition balance in information processing and social dysfunction. Nature 477, 171–178 10.1038/nature10360 21796121 PMC4155501

[B105] Zecevic N. Hu F. Jakovcevski I. (2011). Interneurons in the developing human neocortex. Dev. Neurobiol. 71, 18–33 10.1002/dneu.20812 21154907 PMC3117059

[B106] Zipancic I. Calcagnotto M. E. Piquer-Gil M. Mello L. E. Alvarez-Dolado M. (2010). Transplant of GABAergic precursors restores hippocampal inhibitory function in a mouse model of seizure susceptibility. Cell Transplant. 19, 549–564 10.3727/096368910X491383 20144261

